# Relationships between Cargo, Cell Penetrating Peptidesand Cell Type for Uptake of Non-Covalent Complexes into Live Cells

**DOI:** 10.3390/ph6020184

**Published:** 2013-02-06

**Authors:** Andrea-Anneliese Keller, Franziska Mussbach, Reinhard Breitling, Peter Hemmerich, Buerk Schaefer, Stefan Lorkowski, Siegmund Reissmann

**Affiliations:** 1 Biological and Pharmaceutical Faculty, Friedrich Schiller University, Dornburger Str. 25, 07743 Jena, Germany; 2 Jena Bioscience GmbH, Loebstedter Str. 80, 07749 Jena, Germany; 3 Leibniz Institute for Age Research - Fritz Lipmann Institute, Beutenbergstr. 11, 07745 Jena, Germany

**Keywords:** Cell penetrating peptides, CPPs, formation of non-covalent complexes, MPG peptides, internalized amount of cargoes, relationships between CPP, cargo and cell type.

## Abstract

Modulating signaling pathways for research and therapy requires either suppression or expression of selected genes or internalization of proteins such as enzymes, antibodies, nucleotide binding proteins or substrates including nucleoside phosphates and enzyme inhibitors. Peptides, proteins and nucleotides are transported by fusing or conjugating them to cell penetrating peptides or by formation of non-covalent complexes. The latter is often preferred because of easy handling, uptake efficiency and auto-release of cargo into the live cell. In our studies complexes are formed with labeled or readily detectable cargoes for qualitative and quantitative estimation of their internalization. Properties and behavior of adhesion and suspension vertebrate cells as well as the protozoa *Leishmania tarentolae* are investigated with respect to proteolytic activity, uptake efficiency, intracellular localization and cytotoxicity. Our results show that peptide stability to membrane-bound, secreted or intracellular proteases varies between different CPPs and that the suitability of individual CPPs for a particular cargo in complex formation by non-covalent interactions requires detailed studies. Cells vary in their sensitivity to increasing concentrations of CPPs. Thus, most cells can be efficiently transduced with peptides, proteins and nucleotides with intracellular concentrations in the low micromole range. For each cargo, cell type and CPP the optimal conditions must be determined separately.

## 1. Introduction

Usually cell membranes are permeable only to small and/or hydrophobic molecules. To transport other compounds into live cells, viral vectors are used as well as other robust techniques such as electroporation, magnetofection, and lipofection. Internalization of cargoes for medical purposes requires more subtle methods such as the application of nanoparticles and cell penetrating peptides. In particular, the formation of non-covalent complexes between cell penetrating peptides (CPP) and cargo meets the requirements for diagnostic and therapeutic aims. Procedures utilizing non-covalently complexed CPPs are relatively easy to handle. The complexes are formed by mixing the individual components and the cargo is released after uptake in the cell by dissociation [1,2].

Yet CPPs are not the philosopher’s stone for internalization of cargoes into live cells. For each cargo, cell type, and CPP, optimal conditions must be individually determined. The aim of our study is to investigate the formation of non-covalent complexes of CPPs with different cargoes and the suitability of these complexes for selected cell types. In our studies we characterized the amphiphilicity, hydrophobicity, and proteolytic stability of selected CPPs, and their transport efficiency into certain cell types. The focus of our work is to elucidate the relationships between the chemical properties of the CPP, the properties of the cargo and the cell type, as well as the efficiency of transport and cytotoxicity.

**Figure 1:**
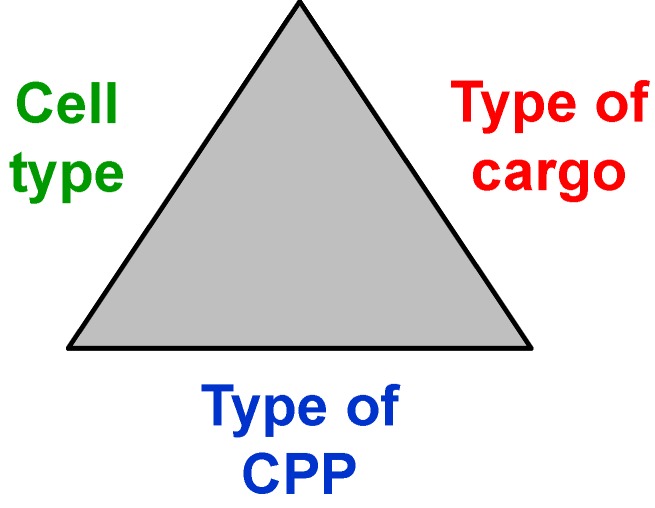
Relationships between Cells, Cargoes and CPPs. The scheme indicates the relationships between the different cell types, cargoes and CPPs. The conditions for each transduction task must be optimized individually.

To investigate these relationships widely used CPPs such as HIV-TAT and penetratin [3,4] were investigated, as well as designed CPPs, such as MPGα, MPGβ, CAD-2 [5,6,7] and the pentapeptide CPPP-2 [8,9]. Uptake efficiencies and cytotoxicity were studied for adhesion cells of different origin, leukemic suspension cells and the protozoa *Leishmania tarentolae* [10]. For elucidating the influence of the cargo on uptake efficiency and cytotoxicity peptides and proteins differing in surface charge and molecular weight between 1–540 kDa were used as cargoes. Furthermore, we studied the uptake of negatively charged, low molecular weight nucleoside triphosphates.

## 2. Results and Discussion

### 2.1. Cell Penetrating Peptides, Cargoes and Cells

For our study we selected the following peptides and proteins from the large number of described molecules with cell penetrating activity as listed in Table 1.

**Table 1:** Cell penetrating peptides used in the presented study.

Amphiphilic-cationic peptides (transcription factors and -activators)		
Penetratin (Antennapedia)	RQIKIWFQNRRMKWKK	8 positive charges, MW 2247 Da
HIV-TAT (47-57)	YGRKKRRQRRR-amide	9 positive charges, MW 1560 Da
MPG peptides		
MPGα	Ac-GALFLAFLAAALSLMGLWSQPKKKRKV-NH-CH_2_-CH_2_-SH	5 positive charges, MW 3047 Da
MPGβ	Ac-GALFLGFLGAAGSTMGAWSQPKKKRKV-NH-CH_2_-CH_2_-SH	5 positive charges, MW 2910 Da
CAD-2 (des-acetyl, Lys^19^-CADY)	GLWRALWRLLRSLWRLLWKA-NH-CH_2_-CH_2_-SH	6 positive charges, MW 2653 Da
Cell penetrating pentapeptide		
CPPP-2	KLPVM	2 positive charges, MW 605 Da
Histone		
Calf thymus histone, type II-AS	more than 100 amino acids	many positive charges, resulting from Arg and Lys residues (more than 20)

Penetratin, and HIV-TAT have been successfully used for the formation of non-covalent complexes [11,12,13,14]. Pep, MPG, and CADY peptides were designed by Heitz and Divita for this purpose over a period of about two decades starting with Pep-1[15]. The labs in Montpellier developed primary and secondary amphipathic peptides [5,6,7], studied conformation of these peptides including conformational flexibility [6,16,17], and mechanism of internalization [7,17,18]. CPPP-2 was used for comparison with the BAX inhibitory peptides and has also cell penetrating properties [8,9]. The lab of Ulo Langel developed peptides from the PepFect family [19,20,21], and used them also for the formation of non-covalent complexes, mainly for transporting splice correcting oligonucleotides. The numerous other cell penetrating peptides were mostly used in covalently bound form, conjugated or fused to the cargo. The peptides MPGα, MPGβ, penetratin and HIV-TAT contain a nuclear localization sequence, enabling the transport of cargoes into the nucleus. The complexes are usually formed in a molar ratio of 1:10, *i.e.* one molecule cargo is complexed with ten CPP molecules. For nucleotides and nucleic acids a fourfold excess of positive charges of the CPP compared to the negative charges of the cargo are used; but CPPP-2 requires a ratio of 1:100 [8]. Histones are also able to transport cargoes into live cells [22]. They are positively charged, direct cargoes into the nucleus and are only moderately cytotoxic [23]. From a practical point of view an optimized mixture of different CPPs has advantages over the use of single peptides. The cocktails allow a universal approach for cargo internalization through compatibility with numerous cell types and triggering different uptake mechanisms.

To investigate the relationships between cargo and CPP we used cargoes of varying structural types such as peptides, proteins and nucleotides. These differed substantially in size and charge. The effectively transported cargoes used in this study are listed in Table 2.

**Table 2:** Cargoes used in different studies.

**Proteins**	
β-Galactosidase	MW = 540 kDa present study and [24]
Bovine serum albumin, ATTO488-labeled	MW = 68 kDa present study and [24]
**Antibodies**	
Antibody anti PI3-kinase-γ, monoclonal, unlabeled	MW ≈ 150 kDa not shown
Antibody anti actin, monoclonal, ATTO488-labeled	MW ≈ 150 kDa not shown
Antibody polyclonal, goat anti mouse, FITC-labeled	MW ≈ 150 kDa [24]
**Peptides**	
Backbone cyclic phosphotyrosine octapeptides	MW ≈ 1.1 kDa only functionally characterized [25]
**Nucleotides **	
Deoxy nucleoside triphosphate, ATTO-labeled ATTO488-dUTP	MW = 1 kDA
	4 negative charges [26]

To investigate the suitability of CPPs for particular cells, different adhesion and suspension cell lines as well the protozoa *Leishmania tarentolae* were used as shown in Table 3. 

**Table 3:** Cell lines used in this study.

**Adhesion cell lines**	
HeLa	Human cervix carcinoma
COS-7	African green monkey kidney
NIH-3T3	Swiss mouse embryo
**Suspension cell lines**	
Jurkat	Human T cell leukemia
NB-4	Human acute promyelocytic leukemia
Kasumi-1	Human acute myeloid leukemia
**Protozoa**	
*Leishmania tarentolae*	Lizard protozoa

The cell lines were obtained from different sources. The strain of *Leishmania tarentolae* was isolated from a lizard. It is non-pathogenic for mammalians and used as a recombinant host for eukaryotic protein expression [10,27].

### 2.2. Proteolytic Activities of Certain Cells and Stabilities of CPPs

**Figure 2:**
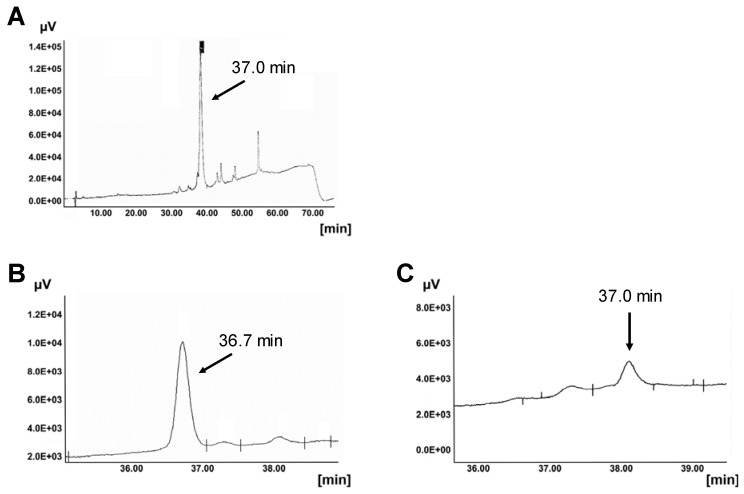
Proteolytic degradation of MPGβ by COS-7 cells. A: MPGβ in the absence of cells. HPLC elution profiles of MPGβ after incubation with COS-7 cells were obtained under described conditions (gradient 10 to 90% acetonitrile). Cleavage of MPGβ by secreted and surface bound proteases was measured after 15 min (B) and 60 min (C) of incubation in the presence of intact cells.

**Figure 3:**
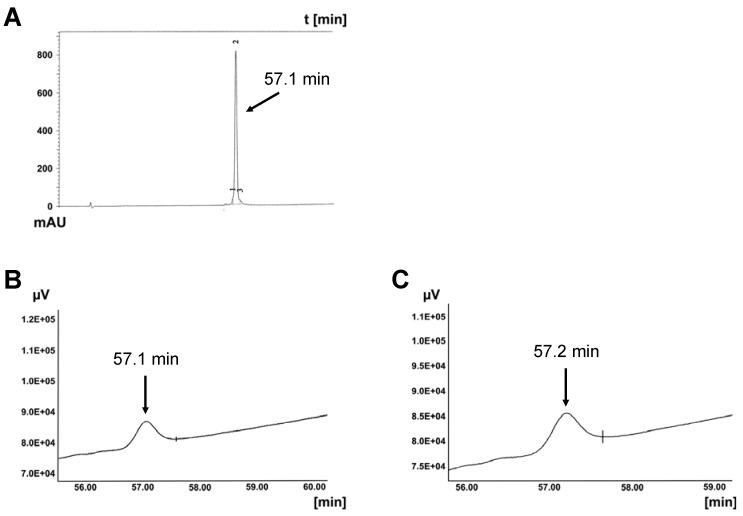
Stability of CAD-2 against proteolytic cleavage by COS-7 and NB-4 cells. HPLC elution profiles were obtained under described conditions (gradient 20 to 90% acetonitrile). A: CAD-2 in the absence of cells. CAD-2 remains stable after 60 min incubation with intact COS-7 **(B)** and NB-4 **(C)** cells.

Chosen CPPs have different chemical properties, *i.e.* distribution of charged, polar and non-polar residues, leading to different amphiphilicity and hydrophobicity. CPPs are degradable by proteases Thus, *Leishmania* cells fully degraded penetratin within 60 min [28]. Mammalian cell lines can also degrade CPPs. HeLa and NIH-3T3 cells cleaved penetratin within 60 min. Their membrane-bound and secreted proteases show strong activity. COS-7 and NB-4 cells were less proteolytic active (data not shown). As shown in Figure 2 the HPLC peak of MPGβ is strongly reduced within 60 min in the presence of COS-7 cells, while CAD-2 remained nearly unaffected by most of the cells investigated (Figure 3). Summarizing, in our experiments penetratin is the most labile CPP, CAD-2 is the most hydrophobic and most stable one.

### 2.3. Transport Efficiencies of CPPs

#### 2.3.1.Dependence on Cell Line

*HeLa cells:* As shown in our preceding publication [24], the CPP cocktail JBS-Proteoducin can internalize enzymatic active β-galactosidase into all cell types investigated, including difficult to transfect Kasumi-1 cells. The active enzyme was transported not only into all types of cells but all of the observed cells are also positively stained using the β-galactosidase substrate 5-bromo-4-chloro-indolyl-β-D-galactopyranoside (X-Gal). The following rank order for internalization into HeLa cells was found: JBS-Proteoducin > MPGα ≥ MPGβ > CAD-2 >> CPPP-2 // HIV-TAT, penetratin [24]. 

**Figure 4:**
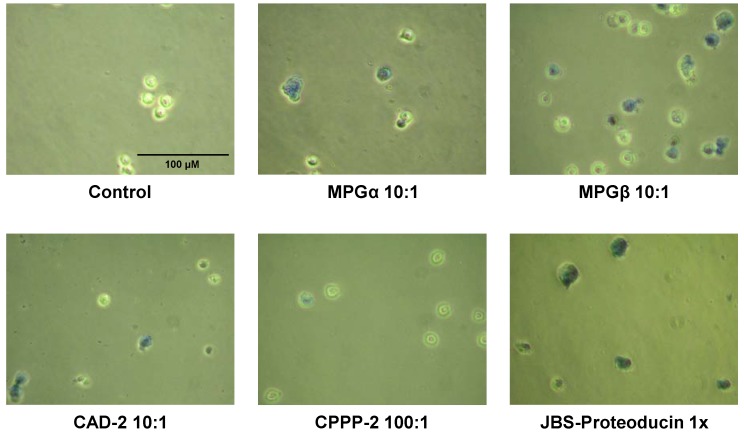
Internalization of β-galactosidase into Jurkat cells using different CPPs.Complexes of β-galactosidase with CPPs formed in the indicated ratios were internalized into Jurkat cells. After staining with X-gal the number of stained cells and the intensity of the staining indicate the efficiencies of the respective CPP.

*Jurkat cells:* Figure 4 shows the different transport efficiencies of the CPPs used for transduction of Jurkat cells. Uptake of β-galactosidase into this suspension cell line occurs with highest efficiency with the CPP cocktail JBS-Proteoducin. Using the β-galactosidase substrate X-Gal all observed cells are intensively stained. For the ratio of 1:10 of the complexes of β-galactosidase and the respective CPP the following rank order was found: JBS-Proteoducin > MPGβ > MPGα > CAD-2 >> CPPP-2. This order is very similar to that for the internalization into HeLa cells as outlined above.

**Figure 5:**
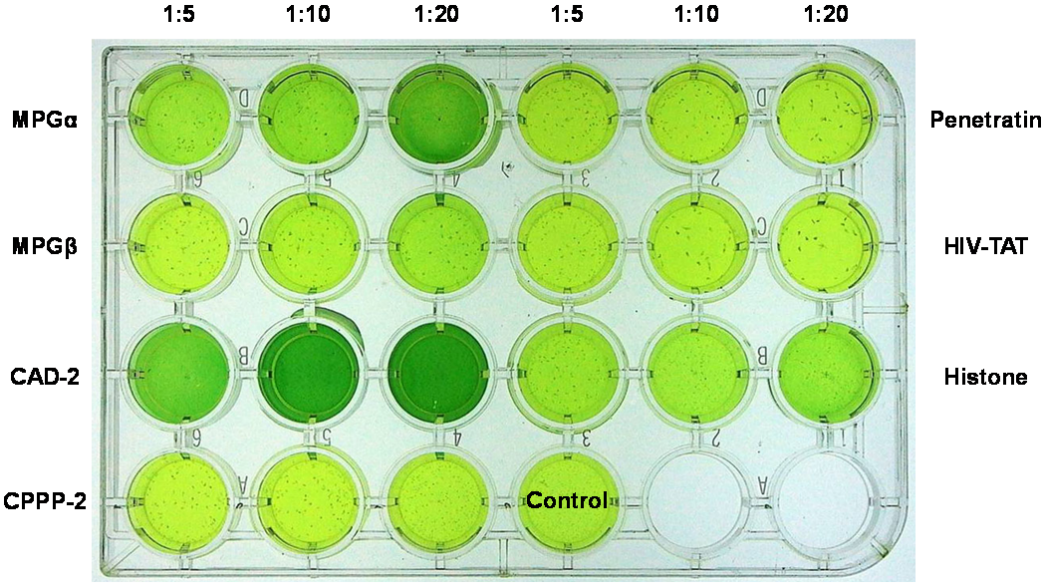
Internalization of β-galactosidase into *Leishmania tarentolae*. Complexes of β-galactosidase with CPPs as well as calf thymus histone formed in the indicated ratios were internalized into *Leishmania*. After staining with X-gal the intensity of the color in the current vial correlates with the intracellular amount of the active enzyme and thus with the transport efficiency of the respective CPP and histone.

*Leishmania tarentolae*: Internalization of the same cargo, β-galactosidase, into live *Leishmania tarentolae* cells is shown in Figure 5. We analyzed not only different CPPs, but also three different molar ratios (1:5, 1:10, 1:20) of cargo and CPP. Intensity of the staining with X-gal and number of stained cells indicates uptake efficiency. In contrast to both mammalian cell lines, HeLa and Jurkat, a different rank order for the uptake efficiency was found for *Leishmania tarentolae*: CAD-2 > MPGα > Histone > MPGβ >> penetratin ≥ HIV-TAT ≥ CPPP-2. With respect to the cargo uptake, these differences between the different cell types most likely result from different membrane compositions and cell characteristics. Enhancing the molar ratios in favor of the CPP can significantly increase the uptake of cargoes even for CPPs which form only weak complexes or exhibit only low uptake efficiencies; this has been observed, for example, for inefficient CPPs such as histone and MPGβ. Despite very similar amino acid sequences, both MPGα and MPGβ show pronounced differences in uptake efficiencies. Because MPGβ is slightly more hydrophilic than MPGα this difference may result from reduced hydrophobic interactions during complex formation. On the other side, MPGβ and hydrophobic CAD-2 transport ATTO488-labeled BSA with lower efficiency into *Leishmania* than MPGα [28], indicating weaker interaction with the cell membrane resulting in a weaker uptake of the CPP-cargo complex. As shown by several authors [5,6,16,17], beside distribution of charge and hydrophobicity within the peptide also conformational shape and flexibility are important for complex formation and cellular uptake. Thus, our results provide evidence for the complexity of complex formation and uptake process and the need for investigating each step separately.

#### 2.3.2. Dependency on the Type of Cargo

Formation of non-covalent complexes between cargo and CPP occurs by optimized interaction of the CPP with the surface of the cargo. These complexes are stabilized by ionic, polar, π-electron-cation, or hydrophobic interactions, hydrogen bonds and common interactions between molecules. Thus, for the same cell type different cargoes show different rank orders for CPPs. Since β-galactosidase is transported into *Leishmania* with the above given rank order, for ATTO488-labeled bovine serum albumin (BSA) another rank order was found which is MPGα >> CAD-2 > histone [28].

A largely different ranking of uptake efficiencies of β-galactosidase was observed for the nucleoside triphosphate ATTO488-dUTP. This nucleotide with a molecular weight of about 1 kDa and four negative charges prefers CPPs in the following rank order: JBS-Nucleoducin > CAD-2 > MPGα ≥ MPGβ > penetratin >> CPP-2. HIV-TAT is not able to transport this nucleotide into HeLa cells [26]. The rank order for the uptake of β-galactosidase into HeLa cells: JBS-Proteoducin > MPGα ≥ MPGβ > CAD-2 >> CPPP-2 // HIV-TAT = penetratin provides evidence for different requirements of enzyme and nucleotide for complex formation and thus for uptake efficiency. Interestingly, the hydrophobic peptide CAD-2 shows higher transport efficiency for the negatively charged nucleotide than the positively charged peptides HIV-TAT and penetratin.

### 2.4. Intracellular Localization

**Figure 6:**
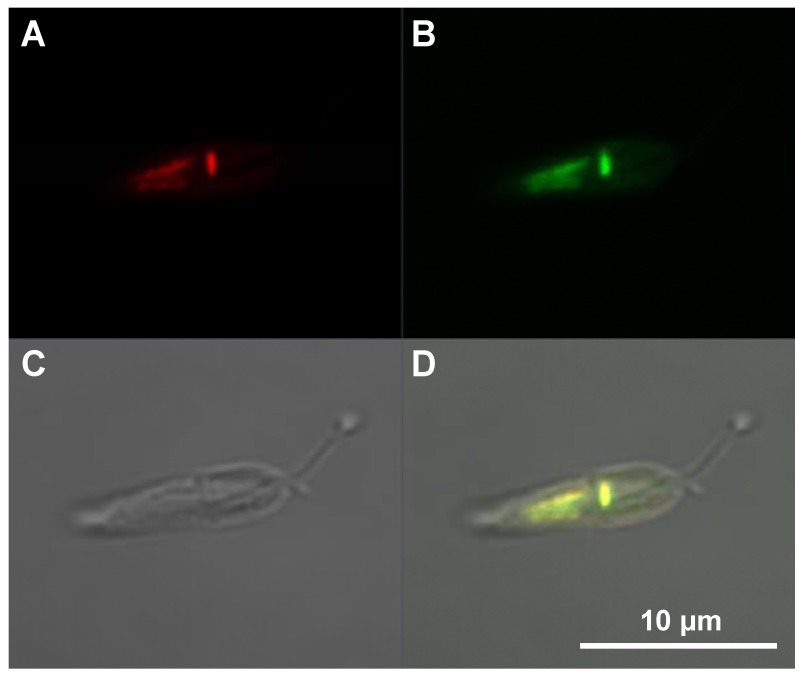
Transduction of ATTO488-BSA into *Leishmania tarentolae* using MPGα. Live cell imaging of the internalized cargo was performed by laser-scanning confocal fluorescence microscopy. DAPI was used for staining of double-stranded DNA (false-colored in red). Colocalization of ATTO488 and DAPI fluorescence is found in the kinetoplast and the nucleus, indicating that MPGα transports ATTO488-BSA into both cell organelles. The fluorescence images represent a mid-nuclear confocal section. A: DAPI staining of cellular DNA (false-colored in red), B: ATTO488-BSA, C: Differential interference contrast, D: Merge.

Using fluorescently labeled cargoes, both internalization and intracellular distribution can be detected by fluorescence microscopy. ATTO488- and FITC-labeled polyclonal antibodies were detected in HeLa cells in vesicles, in the cytosol and in the nuclei following MPGα-mediated uptake [unpublished results]. After internalization of ATTO488-BSA into *Leishmania* using MPGα, the fluorescence was concentrated within the kinetoplast and the nucleus. As seen in Figure 6 ATTO-fluorescence is co-localized with DAPI-staining for double-stranded DNA. The transport into DNA-containing cell organelles occurs in this particular case without any auxiliaries or vesicle destabilizers. Under the same conditions other CPPs showed significantly lower or no fluorescence in *Leishmania* [28]. 

### 2.5. Quantification of Transport Efficiencies

Signaling pathway studies and signal transduction therapy require internalization of sufficient amounts of cargoes into cells. Therefore we were interested in estimating intracellular amounts and concentrations of different cargoes. Using fluorescently labeled cargoes we quantified the intracellular amounts using fluorescence spectroscopic measurements in the case of nucleotides [26], SDS-PAGE in the case of antibodies and BSA [24], and fluorescence intensity for *Leishmania* [28]. The results are listed in Table 4. For unlabeled antibodies the results are in good agreement with Western blot analyses. The internalized amount of ATTO488-BSA was approximately between 20 to 50 attomole per cell. Thus, two to five million molecules were transported into a single HeLa cell. The intracellular concentrations were in the low micromolar range for all types of cells and cargoes. This suggests that the amounts and concentrations are likely sufficiently high to result in complete inactivation of signaling proteins by antibodies.

**Table 4:** Intracellular amounts and concentrations. Internalized amounts are calculated from fluorescence intensities in the homogenate directly or after separation by SDS-PAGE. In the case of nucleoside triphosphates quantification was performed by fluorescence spectrometry. Estimation of cell volumes allows the calculation of intracellular concentrations. The mean volume of HeLa cells is calculated to 1.2 × 10^−5^ µL, that of *Leishmania tarentolae* to 2.0 × 10^−7^ µL. Intracellular amounts are in the attomolar range, intracellular concentrations reach the low micromolar range.

Amount of added complex per 1.6 mL serum-free medium	Internalized amount (0.3 x 10^6^ HeLa cells per well)	
	amol per cell	Intracellular concentration in µM
**ATTO488-deoxy uridine triphosphate into HeLa cells [26]**		
1 µg + JBS-Nucleoducin, charge by charge 1:4	1.1	0.1
5 µg + JBS-Nucleoducin, charge by charge 1:4	2.1	
**ATTO488-labeled bovine serum albumin into HeLa cells [24]**		
10 µg + JBS-Proteoducin, molar ratio 1:10	20	
25 µg + JBS-Proteoducin, molar ratio 1:10	50	4.3
**FITC-antibody (secondary) into HeLa cells [24]**		
5 µg + JBS-Proteoducin, molar ratio 1:10	0.4	
10 µg + JBS-Proteoducin, molar ratio 1:10	2.2	
25 µg + JBS-Proteoducin, molar ratio 1:10	4.3	0.6
**ATTO488-labeled bovine serum albumin into *Leishmania tarentolae***		
1 µg + MPGα, molar ratio 1:10	0.8 x 10^−2^	0.04
5 µg + MPGα, molar ratio 1:10	0.7 x 10^−2^	0.1
10 µg + MPGα, molar ratio 1:10	3.3 x 10^−2^	0.2

### 2.6. Cytotoxicity

To achieve sufficiently high intracellular concentrations of cargoes using non-covalent complexes a ten times higher concentration of CPPs is required. As high CPP concentrations may be cytotoxic, cytotoxicity has to be determined for each CPP. We therefore investigated the influence of increasing concentrations of CPPs on cell viability and membrane integrity.

**Figure 7:**
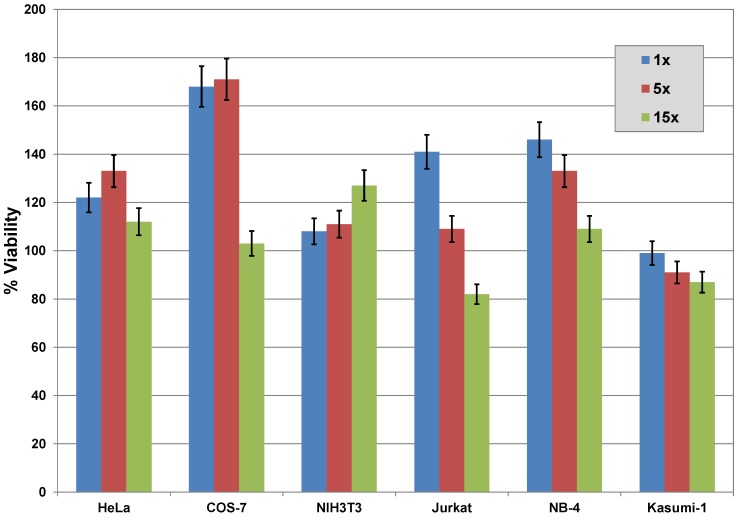
Influence of increasing concentrations of JBS-Proteoducin on viability of different cell lines. Viability of different adhesion and suspension cell lines was measured by colorimetric tetrazolium (MTT) assay. The CPP cocktail is used as recommended one-fold, five-fold, and 15-fold (15×) higher concentration. Cell lines show different sensitivity to JBS-Proteoducin. Cell viability is reduced only marginally, even in high concentrations. Except for Kasumi-1 cells the CPP cocktail enhances cell viability in all cell lines at commonly used concentrations.

Commonly, cell viability is measured via the activity of mitochondrial dehydrogenase using the colorimetric tetrazolium (MTT) assay [29]. Figure 7 shows that the CPP cocktail JBS-Proteoducin reduces viability of all tested cell lines only marginally, even in 15-times higher concentrations than recommended in the supplier’s instructions. The viability of some cell lines is even enhanced, probably by stabilization of mitochondria. As published previously [20,26], single CPPs reduce the viability only in concentrations higher than 10 µM. Because of the low molecular weight and the multiple charges of nucleotides, their transport requires higher concentrations of CPPs or of the peptide cocktail JBS-Nucleoducin, leading to a distinct reduction in cell viability. Thus, the cocktail JBS-Nucleoducin should be used only at recommended concentrations [26]. 

**Figure 8:**
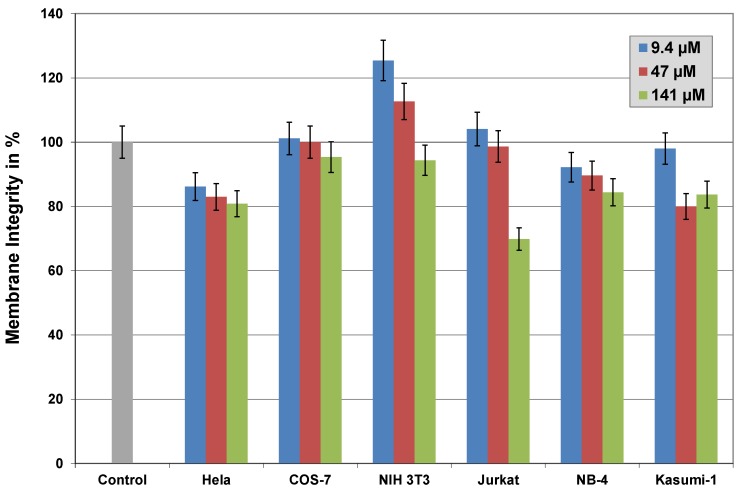
Influence of increasing concentrations of CAD-2 on membrane integrity of different cell lines. Membrane integrity was measured by the release of dead–cell protease using the CytoTox-Glo assay. HeLa and NB-4 cells are sensitive even to low concentrations (9.4 µM). 15-fold higher concentrations of CAD-2 enhance this effect only marginally. For other cell lines the initial value is reduced with increasing concentrations. However, the reduction of membrane integrity with increasing concentrations of CAD-2 up to 141 µM is very low.

Membrane integrity was measured by release of the dead-cell protease into the medium [30], or by FACS analyses with propidium iodide (data not shown). Both tests revealed similar results and both are less sensitive than the MTT assay. But, Figure 8 shows that HeLa and NB-4 cells are sensitive to CPPs even in low concentrations. Because 15-fold higher concentrations of CAD-2 enhanced this effect only marginally, the reduction seems to be a more general effect or methodological problem. For other cell lines the initial value is reduced by increasing concentrations. However, the reduction in membrane integrity is very low. Thus, the measurement of membrane integrity is much less sensitive to increasing concentrations of CPPs than the MTT assay. 

Cytotoxic effects in *Leishmania* were estimated by the influence of CPPs and cocktails on morphology and motility of the cells. *Leishmania tarentolae* cells were already very sensitive to MPGα and MPGβ at concentrations of 10 µM. These peptides act most likely as membrane active anti-microbiotics destroying the structure of the cell membrane. While *Leishmania* cells were more sensitive to MPG peptides than mammalian cells, they were less sensitive than mammalian cells to some other CPPs as shown in Table 5.

**Table 5:** Cytotoxicity of cell penetrating peptides and CPP cocktails *in Leishmania tarentolae.*

**Amphiphilic-cationic peptides (transcription factors and -activators)**	
Penetratin (Antennapedia)	≤ 10 µM
HIV-TAT (47-57)	≥ 30 µM
**MPG peptides**	
MPGα	≤ 5 µM
MPGβ	≤ 5 µM
CAD-2	≤ 25 µM
**Cell penetrating pentapeptide**	
CPPP-2	≥ 100 µM
**Histone **	
Calf thymus histone, type II-AS	≈ 5 µM
**Cocktails**	
JBS-Proteoducin	≥ 50-fold of recommended amount
JBS-Nucleoducin	≥ 30-fold of recommended amount

## 3. Experimental

### 3.1. Materials

*Cell Penetrating Peptides and CPP Cocktails JBS-Proteoducin and JBS-Nucleoducin*: MPGα, MPGβ, CAD-2, penetratin, HIV-Tat (47-57), CPPP-2, JBS-Proteoducin, JBS-Nucleoducin and auxiliaries were obtained from Jena Bioscience (Jena, Germany), histone from calf thymus, type II-AS from Sigma-Aldrich Chemie (Taufkirchen, Germany).

*Cells*: HeLa, COS-7, NIH-3T3, Jurkat, NB-4, and Kasumi-1, *Leishmania tarantolae* cells (LEXSY host P10) were obtained from Jena Bioscience and cultivated under recommended conditions.

*Cargoes and Reagents:* Fluorescent-labeled nucleotide: Aminoallyl ATTO488-dUTP, (Jena Bioscience), fluorescent-labeled antibodies: polyclonal, goat anti mouse, FITC-labeled (Jena Bioscience), fluorescence-labeled BSA (ATTO488-BSA, Jena Bioscience), β-galactosidase (*E. coli*, 540 kDa, Calbiochem, Darmstadt, Germany), X-gal (99 %, Calbiochem), trypsin/EDTA solution (trypsin/EDTA concentrate, PAA, Pasching, Austria), DAPI (Sigma-Aldrich Chemie), 1 % penicillin/streptomycin and Hemin (Jena Bioscience).

*Kits*: Cell viability assay: MTT assay (Jena Bioscience), membrane integrity assay: bioluminescence test CytoTox-Glo (Promega, Madison, WI, USA), Mycoplasma test (Mycoplasma Detection Kit, Jena Bioscience). 

*Buffers and Solvents:* Tris Pufferan, Hepes Pufferan (Carl Roth, Karlsruhe, Germany), Dulbecco’s phosphate buffer solution 1x (PBS) without Ca^2+^ and Mg^2+^, pH 7.0 - 7.5 (PAA), glycine buffer, pH 3.0, 200 mM (Jena Bioscience), DMSO (Jena Bioscience), LEXSY BHI (Jena Bioscience). 

### 3.2. Equipment

Analytical high performance chromatograph JASCO PU-987 (JASCO, Gotha, Germany), centrifuge Eppendorf 5424 (Eppendorf, Hamburg, Germany), Nikon Eclipse TS100 microscope (Nikon, Düsseldorf, Germany), Axiophot fluorescence microscope (Carl Zeiss, Oberkochen, Germany), LSM 710 laser scanning confocal microscope (Carl Zeiss, Jena, Germany), fluorescence spectrometer LS50 (Perkin Elmer, Waltham, MA, USA), UV/VIS spectrometer Lambda 35 (Perkin-Elmer), luminescence plate reader Fluostar OPTIMA (BMG Labtech, Offenburg, Germany).

*Electrophoresis*: Vertikal Mini Polyacrylamide Gel System (Biorad, Munich, Germany), protein-gel loading buffer Roti®-Load 2, non-reductive (Carl Roth), fluorescence densimeter: Digital fluorescence densitometer (UV Transimulator and CCD Video Camera Modul, LTF Labortechnik, Wasserburg, Germany).

### 3.3. Methods

#### 3.3.1. Degradation of CPPs by Proteases

In a 6-well-plate with 0.3 × 10^6 ^cells per well the different cell types were cultivated overnight under optimized conditions. Then medium was removed by aspiration (adherent cell) or centrifugation (suspension cells) and washed cells were incubated with CPPs in phosphate buffer solution (PBS). Incubation was performed under the same conditions as the transduction of complexes with the cargo for 1 h at 37 °C in a humidified atmosphere containing 5% CO_2._ After 15, 30, 45 and 60 min samples of 200 µL were taken and the proteolytic reaction was stopped by addition of 50 µL 37 perc. HCl. After freezing and lyophilization samples were analyzed by analytical high performance liquid chromatography (HPLC), using a RP_18_ column (Vydac 218TP). In case of MPGβ, for elution a gradient was used from 10% acetonitrile and 90% water, both containing 0.1% trifluoroacetic acid to 60% acetonitrile and 40% water in 50 min. Because CAD-2 is more hydrophobic the gradient started at 20% acetonitrile and increases in 60 min to 80% acetonitrile. The flow rate was 1.0 ml/min and detection was performed at 220 nm.

#### 3.3.2. Preparation of Stock Solutions

CPPs (0.5 mg) or the whole content of the vials with JBS-Proteoducin or JBS-Nucleoducin were dissolved in 1.25 to 1.50 mL of sterile and oxygen free water. To obtain ten-times higher molar ratio with CPPP-2, 1.2 mg were dissolved in 1 mL oxygen-free water. The solution was roughly mixed, frozen to -80 °C and thawed (repeated three times), and sonicated for 5 min. The obtained stock solutions were used immediately or stored as aliquots at -20 °C.

#### 3.3.3. Complexing Cargoes with Peptides and Proteins

A detailed description of formation of complexes with peptides and proteins is given in a previous publication [18]. Briefly, cargo proteins (ATTO488-BSA, MW ≈ 68 kDa, β-galactosidase, MW ≈ 540 kDa) and the calculated volume of stock solution of CPPs or JBS-Proteoducin were dissolved separately in 100 µL of 1× PBS). Both solutions were thoroughly mixed by repeated pipetting. The mixture was incubated for 30 minutes at 37 °C to achieve complex formation. The molar ratio of cargo to CPPs was usually calculated to 1:10. For internalization of higher amounts of cargo, both the amount of protein and stock solution of CPP or cocktail, respectively, were multiplied. Complexes of the enzyme β-galactosidase and JBS-Proteoducin were formed from 1 µg β-galactosidase and 1 µL stock solution of JBS-Proteoducin under the described conditions.

#### 3.3.4. Internalization

Cells were cultivated and transduced under commonly used conditions. Only low passage numbers were used and the cells were checked microscopically for their vital shape and absence of bacteria. Additionally, cells were checked for absence of mycoplasma by PCR. A solution of penicillin and streptomycin was added to prevent growth of bacteria and fungi during incubation.

##### 3.3.4.1. Internalization of β-Galactosidase and Antibodies into Jurkat Cells

The thoroughly washed cell pellet was suspended in 200 µL of complex solution followed by 400 µL of serum free medium, gently mixed and transferred into 24-well plates. Incubation was started for 1 h at 37 °C in a humidified atmosphere containing 5% CO_2_. After addition of 1.4 mL of complete culture medium incubation was continued at 37 °C for approx. 2 h in a humidified atmosphere containing 5% CO_2_. The cells were washed by spinning down twice with PBS, three times with glycine buffer (pH 3.0) and twice with PBS.

##### 3.3.4.2. Cultivation and Transduction of *Leishmania tarentolae*

*Leishmania* cells were cultivated as described previously [21,22]. They were grown as suspension culture at 26 °C in complex medium (LEXSY BHI) completed with Hemin (final concentration of 5µg/mL), penicillin and streptomycin. 10 ml of stock cultures with an optimum OD of 1.4-1.8 (ca. 5-8 x 10^7^ cells/mL) were subcultivated twice per week at 1:20 and 1:50 ratios.

For transduction cells in an early stage of development were used. To this end stock cultures were diluted 1:10 the day before the experiment into 10 mL of culture medium in 25 cm^2^-ventilated tissue culture flasks and were incubated overnight in the dark at 26 °C for optimal proliferation. Cell suspensions with an OD of 1.2 (ca. 4 × 10^7^ cells/mL) were used for transduction. The cells were thoroughly washed twice with PBS (5 mL each) by centrifugation (2,000 × g, 3 min). Pellets were resuspended in the original volume of PBS containing 0.1% BSA and aliquoted into 24-well plates. Four hundred µL of cell suspension were used per experiment. Two hundred µL of the complex solutions were added to these cells and mixtures were incubated for 1 h at 26 °C in the dark with gentle agitation. Successively, 1 mL of complete growth medium (LEXSY BHI) was added per sample and incubation was continued at 26 °C for 2 h. Cells were washed twice with PBS (2 mL each) by centrifugation (2,000 × g, 3 min) prior to analysis of transduction.

#### 3.3.5. Staining of Internalized β-Galactosidase

Staining of the cells took place in multiwell-plates. Jurkat and Leishmania cells were stained according to the instructions described in [24], using a staining solution containing potassium ferrocyanide, potassium ferricyanide and X-gal. Leishmania cells were stained after finishing the above described transduction protocol. For Jurkat cells the staining was performed after transferring cells from spinning tubes used in the last washing step to 24-well plates. Cells were incubated in staining solution overnight and directly observed in a microscope using a 400-fold magnification.

#### 3.3.6. Confocal Fluorescence Microscopy

For confocal microscopy of fixed and living cells, a LSM 710 laser scanning confocal microscope was used as previously described [31]. Briefly, samples were scanned using a 63x Plan-Apochromat oil immersion objective. Blue, green and red fluorescent dyes were excited by laser light at 405, 488, 543, and 633 nm wavelengths, respectively. To avoid bleed-through effects in multi-fluorescence staining experiments, each dye was scanned independently in a multitracking mode. Differential interference contrast (DIC) images were acquired with the 488 nm laser line along with scanning green fluorescent dyes.

#### 3.3.7. Calculation of Intracellular Cargo Concentrations

Quantitative fluorescence measurements on internalized fluorescent nucleotide [26] and SDS-PAGE with fluorescent proteins [24] allow the calculation of the internalized amount of cargo. Division of this value by the cell number yields the amount per cell. The diameter of a HeLa cell was estimated from fluorescent microscopic pictures using a calibration scale. The mean volume was calculated to 1.2 × 10^−5 ^µL. The amount of internalized fluorescent cargo divided by the mean cell volume provides the intracellular concentration. Uptake of ATTO488-BSA into *Leishmania tarentolae* was estimated with a fluorescence plate reader after cell lysis [28]. The volume of *Leishmania tarentolae* was calculated to 2.0 × 10^−7^ µL.

#### 3.3.8. Cell Viability Assay

The MTT assay was performed according to the supplier’s instructions. Briefly, cells were cultivated under commonly used conditions and treated in serum-free medium for 1 h at 37 °C with different CPPs or with the cocktail. After removal of CPPs and repeated washings the viability of the cells was estimated by the MTT assay. Untreated cells are defined as 100% viable. The assay was performed according to the instructions in 6-well plates. Each value was estimated as a triplicate with a SD of ± 1.3. The formed formazan was measured with an UV/VIS spectrometer at 508 nm.

#### 3.3.9. Membrane Integrity Assay

Cells were cultured under standard conditions and treated in serum-free medium for 1 h at 37 °C with different CPPs and the cocktail. After removal of CPPs and repeated washings the membrane integrity of the cells was estimated by the bioluminescence test CytoTox-Glo measuring the release of a cytosolic dead-cell protease. The CytoTox-Glo assay was performed according to the supplier’s instructions and adapted to 96-well plates. Bioluminescence was estimated by a luminescence plate reader. Each value was measured in triplicates with an SD of ± 1.2.

#### 3.3.10. Cytotoxicity Test for *Leishmania tarentolae*

*Leishmania* cells were cultivated as described previously, resuspended in PBS and treated with increasing concentrations of cocktails and CPPs for 1 h. Cytotoxic effects were monitored by changes in morphology and motility observed using a microscope at 400-fold magnification.

#### 3.3.11. Mycoplasma Test

The assay was performed according to the supplier’s instructions. This PCR assay is very sensitive and enables an early detection of contamination.

## 4. Conclusions

Internalization of cargoes into live cells using non-covalent complexes with cell penetrating peptides is an easy to handle and subtle process suitable for basic reasearch and therapy. However, CPPs able to transport cargoes into live cells are not the philosopher´s stone. The choice of the right CPP for a given task requires characterization of complex formation with the cargo used, validation of internalization efficiency into the cell type of interest and determination of cytotoxic effects.

Formation of non-covalent complexes between cargoes and CPPs occurs through non-covalent interactions, mainly hydrophobic and ionic binding. Even in the case of nucleoside triphosphate the hydrophobic interaction between aromatic base and hydrophobic residues of CPPs seems to be more important than the formation of ion pairs. Conformational shape and flexibility of the CPPs also influence binding to the cargo and to the cell surface [6,16,32]

CPPs exhibit different stabilities against surface-bound or secreted proteases of the targeted cells. In our experiments CAD-2 was the most hydrophobic and most stable one. Less stable CPPs can be protected from proteolytic degradation by addition of protease inhibitors.

Internalization starts with binding to receptors, proteoglycans [33] or distinct areas of cell membranes formed by micro-heterogeneity [34] and takes place by a complex uptake process [35]. Depending on composition and properties of the cell surface CPPs show different suitability for certain mammalian or protozoa cells.

Cocktails of CPPs have advantages because they trigger different uptake mechanisms and are applicable to a broad spectrum of cells and cargoes. Because of the required tenfold or higher excess of CPPs over the amount of cargo their influence on viability and membrane integrity has to be determined. Both parameters of live cells are only marginally reduced at CPP concentrations up to 10 µM. However, the cell lines studied here differ strongly in their response to higher concentrations of CPPs and CPP cocktails. The intracellular amounts of proteins and nucleotides can reach low micromolar concentrations. Thus, internalized antibodies, for example, allow inhibition of signaling proteins and open a new possibility for investigating signaling processes in competition with siRNA-based knockdown techniques. Internalization of nucleotides, especially GTP analogues, allows studies on GTP-binding proteins, their role and influence in metabolism and signaling pathways in live cells.

## References

[1] Heitz F., Morris M.C., Divita G. (2009). Twenty years of cell-penetrating peptides: from molecular mechanism to therapeutics. Br. J. Pharmacol..

[2] Deshayes S., Morris M., Heitz F., Divita G. (2008). Delivery of proteins and nucleic acids using a non-covalent peptide-based strategy. Advanced Drug Del. Reviews.

[3] Lindgren M., Langel U., Langel U. (2011). Classes and prediction of cell-penetrating peptides. Cell penetrating peptides Methods and Protocols. Methods in Molecular Biology.

[4] Dupont E., Prochiantz A., Joliot A., Langel U. (2011). Penetratin story: An overview. Cell penetrating peptides. Cell penetrating peptides Methods and Protocols. Methods in Molecular Biology.

[5] Deshayes S., Plenat Th., Aldrian-Herrada G., Divita G., Le Grimellec Ch., Heitz F. (2004). Primary amphipathic cell penetrating peptides: Structural requirements and interactions with model membranes. Biochemistry.

[6] Kurzawa L., Pellerano M., Morris M.C. (2010). PEP and CADY-mediated delivery of fluorescent peptides and proteins into living cells. Biochim. Biophys. Acta.

[7] Crombez L., Aldrian-Herrada G., Konate K., Nguyen Qu.N., McMaster G.K., Brasseur R., Heitz F., Divita G. (2009). A new potent secondary amphipathic cell-penetrating peptide for siRNA delivery into mammalian cells. Mol. Therapy.

[8] Gomez J.A., Gama V., Yoshida T., Sun W., Hayes P., Leskov K., Boothman D., Matsuyama S. (2007). Bax-inhibiting peptides derived from Ku70 and cell-penetrating pentapeptides. Biochem. Soc. Trans..

[9] Gomez J., Matsuyama S., Langel U. (2011). Cell-penetrating penta-peptides and BAX-inhibiting peptides: Protocol for their application. Cell penetrating peptides Methods and Protocols. Methods in Molecular Biology.

[10] Breitling R., Klingner S., Callewaert N., Pietrucha R., Geyer A., Ehrlich G., Hartung R., Mueller A., Contreras R., Beverley St.M., Alexandrov K. (2002). Non-pathogenic trypanosomatid protozoa as a platform for protein research and production. Protein Expr. Purific..

[11] Aamand H.L., Norden B., Fant K. (2012). Functionalization with C-terminal cysteine enhances transfection efficiency of cell-penetrating peptides through dimer formation. Biochem. Biophys. Res. Comm..

[12] Richard J.P., Melikov K., Brooks H., Prevot P., Lebleu B., Chernomordik L.V. (2005). Cellular uptake of unconjugated TAT peptide involves clathrin-dependent endocytosis and heparan sulfate receptors. J. Biol. Chem..

[13] Ignatovich I.A., Dishe E.B., Pavlotskaya A.V., Akifiev B.N., Burov S.V., Orlov S.V., Perevozchikov A.P. (2003). Complexes of plasmid DNA with basic domain 47–57 of HIV-1 TAT protein are transferred to mammalian cells by endocytosis-mediated pathways. J. Biol. Chem..

[14] Jeang K.T., Xiao H., Rich E.A. (1999). Multifaced activities of the HIV-1 transactivator of transcription, TAT. J. Biol. Chem..

[15] Gros E., Deshayes S., Morris M.C., Aldrian-Herrada G., Depollier J., Heitz F., Divita G. (2006). A non-covalent peptide-based strategy for protein and peptide nucleic acid transduction. Biochim. Biophys. Acta.

[16] Chaloin L., Vidal P., Heitz A., Van Mau N., Mery J., Divita G., Heitz F. (1997). Conformations of primary amphipathic carrier peptides in membrane mimicking environments. Biochemistry.

[17] Deshayes S., Heitz A., Morris M.C., Charnet P., Divita G., Heitz F. (2004). Insight into mechanism of internalization of cell-penetrating carrier peptide Pep-1 through conformational analysis. Biochemistry.

[18] Morris M.C., Gros E., Aldrian-Herrada G., Choob M., Archdeacon J., Heitz F., Divita G. (2007). A non-covalent peptide-based carrier for *in vivo* delivery of DNA mimics. Nucleic Acid Res..

[19] Mäe M., EL Andaloussi S., Lundin P., Oskolkov N., Johansson H.J., Guterstam P., Langel U. (2009). A stearylated CPP for delivery of splice correcting oligonucleotides using a non-covalent co-incubation strategy. J. Controlled Rel..

[20] Oskolkov N., Arukuusk P., Copolovici D.-M., Lindberg St., Margus H., Padar K., Pooga M., Langel U. (2011). NickFects, phosphorylated derivatives of transportan 10 for cellular delivery of oligonucleotides. Int. J. Pept. Res. Therapeutics.

[21] Ezzat K., EL Andaloussi S., Zaghloul E.M., Lehto T., Lindberg St., Moreno P.M.D., Viola J.R., Magdy T., Abdo R., Guterstam P., Sillard R., Hammond S.M., Wood M.J.A., Arzumanov A.A., Gait M.J., Smith C.I.E., Hällbrink M., Langel U. (2011). PepFect 14, a novel cell-penetrating peptide for oligonucleotide delivery in solution and as solid formulation. Nucleic Acid Res..

[22] Hariton-Gazal E., Rosenbluh J., Graessmann A., Gilon C., Loyter A. (2003). Direct translocation of histone molecules across cell membranes. J. Cell Sci..

[23] Singh R.K., Liang D., Gajjalaiavari U.R., Kabaj M.-H.M., Paik J., Gunjan A. (2010). Excess histone levels mediate cytotoxicity via multiple mechanisms. Cell Cycle.

[24] Mussbach F., Franke M., Zoch A., Schaefer B., Reissmann S. (2011). Transduction of peptides and proteins into live cells by cell penetrating peptides. J. Cell. Biochem..

[25] Zoda M.S., Zacharias M., Mussbach F., Schaefer B., Reissmann S. Assembly and stimulatory activity of backbone to side chain cyclic octapeptide-ligands for the N-terminal SH2-domain of the protein-tyrosine phosphatase SHP-1. Proceedings of the 31^st^ European Peptide Symposium September 5-9, 2010.

[26] Mussbach F., Pietrucha R., Schaefer B., Reissmann S., Langel U. (2011). Internalization of nucleoside phosphates into live cells by complex formation with different cell penetrating peptides and JBS-Nucleoducin. Cell penetrating peptides Methods and Protocols. Methods in Molecular Biology.

[27] LEXSY-Eukaryotic protein expression in *Leishmania tarentolae*. http://www.jenabioscience.com/cms/en/1/browse/1838,.

[28] Keller A.-A., Breitling R., Hemmerich P., Braun M., Schaefer B., Lorkowski S., Reissmann S. Transduction of proteins into *Leishmania tarentolae* by formation of non-covalent complexes with cell-penetrating peptides.

[29] Wu R.P., Youngblood D.S., Hassinger J.N., Lovejoy C.E., Nelson M.H., Iversen P.L., Moulton H.M. (2007). Cell-penetrating peptides as transporters for morpholino oligomers: effects of amino acid composition on intracellular delivery and cytotoxicity. Nucleic Acids Res..

[30] Niles A.L., Moravec R.A., Hesselberth P.E., Scurria M.A., Daily W.J., Riss T.L. (2007). A homogeneous assay to measure live and dead cells in the same sample by detecting different protease markers. Anal. Biochem..

[31] Brand P., Lenser T., Hemmerich P. (2010). Assembly dynamics of PML nuclear bodies in living cells. PMC Biophysics.

[32] Deshayes S., Konate K., Aldrian G., Crombez L., Heitz F., Divita G. (2010). Structural polymorphism of non-covalent peptide-based delivery systems: Highway to cellular uptake. Biochim. Biophys. Acta.

[33] Nakase I., Tadokoro A., Kawabata N., Takeuchi T., Katoh H., Hiramoto K., Negishi M., Nomizu M., Sugiura Y., Futaki S. (2007). Interaction of arginine-rich peptides with membrane-associated proteoglycans is crucial for induction of actin organization and macropinocytosis. Biochemistry.

[34] Foerg C., Ziegler U., Fernandez-Carneado J., Giralt E., Renner R., Beck-Sickinger A., Merkle H.P. (2005). Decoding the entry of two novel cell-penetrating peptides in HeLa-cells: Lipid raft-mediated endocytosis and endosomal escape. Biochemistry.

[35] Madani F., Lindberg S., Langel U., Futaki S., Graeslund A. (2011). Mechanism of cellular uptake of cell-penetrating peptides. J. Biophysics.

